# No evidence for higher rates of hepatocellular carcinoma after direct-acting antiviral treatment: a meta-analysis

**DOI:** 10.20517/2394-5079.2019.19

**Published:** 2019-08-07

**Authors:** Stephanie M. Rutledge, Hui Zheng, Darrick K. Li, Raymond T. Chung

**Affiliations:** 1 Department of Medicine, Massachusetts General Hospital, Gastroenterology Unit/Warren 10, Boston, MA02114, USA.; 2 Liver Center, Gastrointestinal Division, Department of Medicine, Massachusetts General Hospital, Harvard Medical School, Boston, MA 02114, USA.

**Keywords:** Humans, hepatitis C virus, liver cirrhosis, liver neoplasms, interferons

## Abstract

**Aim:**

Hepatitis C virus (HCV) is the leading cause of hepatocellular carcinoma (HCC) in the United States. Achieving sustained viral response with interferon (IFN) treatment reduces the risk from 3%-5% to 0.5%-1% annually. Several studies reported unexpectedly high rates of HCC after treatment with direct-acting antivirals (DAAs). The aim of our study was to compare HCC rates in DAA-, IFN-treated and untreated populations.

**Methods:**

A literature search was conducted using ScienceDirect, Ovid®, Web of Science and MEDLINE through January 2019. Studies were included if they measured rates of *de novo* or recurrent HCC (following curative treatment) in HCV-infected persons. We included 138 studies (*n* = 177,512). Simple pooling of data and meta-analysis were performed, using the random effects method.

**Results:**

Mean age was higher in the DAA-treated *vs.* IFN-treated group (58.4 years *vs.* 52.6 years; *P* = 0.0073), as were diabetes prevalence (34.5% *vs.* 11.7%; *P* ≤ 0.001) and incident cirrhosis (47.8% *vs.* 34.2%, *P* = 0.0017). The incidence rate of *de novo* HCC was 2.01/100 person-years (py) (95%CI: 1.38, 2.67) in the DAA group and 1.45/100py (95%CI: 0.98, 1.94) in the IFN-treated group. HCC recurred at 16.76/100py (95%CI: 10.75, 22.91) in the DAA-treated group *vs.* 20.04/100py (95%CI: 2.58, 45.21) after IFN. After adjusting for factors such as age and cirrhosis, the hazard ratio was 0.58 (95%CI: 0.20, 1.07) for HCC occurrence and 0.59 (95%CI: 0.24, 1.03) for HCC recurrence after DAA treatment compared to IFN-based treatment.

**Conclusion:**

We did not find evidence for increased rates of HCC in DAA-treated compared with IFN-treated patients. Compared to those treated with IFN, older patients with additional risk factors for HCC were treated with DAAs. This imbalance appears to explain the higher numerical incidence of HCC among DAA-treated patients.

## INTRODUCTION

Hepatocellular carcinoma (HCC) is the fastest rising cause of cancer-related death in the United States^[1]^. In most developed countries, chronic hepatitis C virus (HCV) infection is the leading risk factor for HCC. Approximately half of the increase in HCC cases in the United States may be accounted for by the aging cohort with chronic HCV infection^[1]^. Though the presence of cirrhosis is an important risk factor for the development of HCC in HCV-infected individuals, HCV itself may have pro-carcinogenic properties^[2]^. Specifically, the virus induces tumor development indirectly via inflammatory and pro-fibrotic host responses and may also exert direct oncogenic effects upon the infected cell, via deregulation of host cell checkpoints, oxidative stress and DNA damage^[2]^.

Patients with HCV-related cirrhosis have a risk of developing HCC, estimated at 3%-5% per year^[3]^. The risk is further enhanced by alcohol misuse, diabetes mellitus, obesity and coinfection with hepatitis B or HIV^[4,5]^. Studies have shown that achieving sustained viral response (SVR) after interferon (IFN) treatment reduces the risk of HCC to 0.5%-1% per year^[6–8]^. It was originally believed that IFN reduced the risk via antiviral as well as direct anti-tumor effects but non-sustained responders to IFN do not achieve the same reduction in HCC risk^[9]^. This may be related to the fact that IFN delays the development of HCC but does not prevent it in the presence of persistent viremia and cirrhosis.

With the FDA approval of IFN-free regimens in 2014, it was anticipated that the risk of HCC would be further reduced due to their high SVR achievement rates (upwards of 95% compared to approximately 56% with pegylated-IFN and ribavirin regimens)^[10,11]^. Thus it was surprising when several sentinel European reports published in 2016 raised concern for increased rates of HCC in patients treated with IFN-free direct-acting antiviral (DAA) regimens. Most of the studies reported increased rates of HCC recurrence but a few reported increased rates of *de novo* HCC. Some of these tumors were diagnosed within weeks to months of DAA treatment and several studies observed that tumors were unusually aggressive and locally invasive on imaging^[12–15]^. Later, conflicting studies were published that did not show evidence of increased risk of HCC, with follow-up periods of up to 15 months^[16–18]^.

However, all the studies had significant limitations. They were observational in nature, each with small numbers of patients. They evaluated heterogeneous populations with varying numbers of patients with cirrhosis. The question was raised as to whether the perceived increased risk was an artifact of selection bias, whereby older patients with more advanced liver disease and additional risk factors for HCC are being treated with DAA than would historically been treated in the era of IFNs.

The development of a safe, efficacious and well-tolerated treatment has revolutionized the landscape of HCV treatment. Patients treated with DAAs have been shown to have lower rates of decompensation and model for end-stage liver disease (MELD) score progression than those not treated with DAA agents^[16,19]^. Finding a correlation between DAAs and HCC development or recurrence would have major implications. Thus, to find a more definitive answer to this question, we compared rates of HCC occurrence and recurrence in DAA-treated persons with IFN-treated and untreated patients in a meta-analysis of all published studies.

## METHODS

### Data sources

A comprehensive literature search was performed using ScienceDirect, Ovid®, Web of Science, MEDLINE, Google Scholar and the Cochrane Library. Abstract books from the major international hepatology meetings including European Association for the Study of the Liver and American Association for the Study of Liver Diseases were also examined thoroughly for additional studies. We searched databases from inception through January 2019 and included studies with human subjects which measured rates of HCC occurrence or recurrence in persons infected with HCV.

### Study selection

We considered retrospective or prospective observational cohort studies and randomized controlled trials as eligible studies for analysis. We included studies if they assessed (1): *de novo* HCC development in patients with chronic HCV; or (2): HCC recurrence in patients with chronic HCV who had received successful HCC curative treatment and were believed to be cancer-free at the time of HCV treatment. HCC treatments which were categorized as being potentially curative included liver resection, microwave coagulation therapy, percutaneous ethanol injection therapy, radiofrequency ablation, and liver transplantation. We included HCV-infected patients regardless of the presence or absence of cirrhosis and regardless of HCV treatment status (DAA-treated, IFN-treated or untreated). Subjects with and without SVR were included. Where we found multiple studies from the same population, the most recent studies were included. All full text manuscripts and conference abstracts were considered for inclusion. Studies with missing essential data or with unclear or less rigorous methodology were excluded. We excluded studies with a follow-up period of less than 1 year, to avoid including cases where sub-clinical HCC was likely present at the time of treatment initiation. The quality of evidence in each included study was assessed using the Cochrane tool for risk of bias [Supplementary Tables 1 and 2].

### Data extraction

We manually pulled data from studies into a pre-formatted standardized spreadsheet containing clinical, demographic and epidemiological headings. In the spreadsheet, studies were categorized by treatment type (DAA-treated, IFN-treated or untreated) and primary endpoint of HCC occurrence or recurrence. They were then further sub-divided into SVR and non-SVR groups where this information was available from studies.

### Data analysis

The outcomes evaluated were HCC occurrence and HCC recurrence. Studies with zero events were excluded from the analysis. The incidence rates of HCC occurrence or recurrence were calculated per 100py. Meta-analyses, stratified by type of HCV treatment received (DAA, IFN and never treated), were undertaken to determine incidence rates for each group using a random-effects model. Several studies had performed multivariate analysis adjusting for a variety of factors including age, gender, baseline cirrhosis status, baseline alpha fetoprotein (AFP), ethnicity and Child-Pugh score. We used a mixed effects meta-analysis to calculate the overall adjusted and unadjusted hazard ratio of DAA treatment, using studies with multivariate analyses. Several sub-analyses were performed, including exclusion of any HCC event diagnosed within the first six months after the completion of treatment for HCV, and the calculation of the annualized HCC rate for the second year after HCV treatment. In order to obtain this data, we manually extracted information from studies where the time-to-event for each HCC event was recorded.

For baseline characteristics within individual studies, data were weighted, then pooled and *P*-value generated using GraphPad Prism software.

## RESULTS

**Figure 1.**
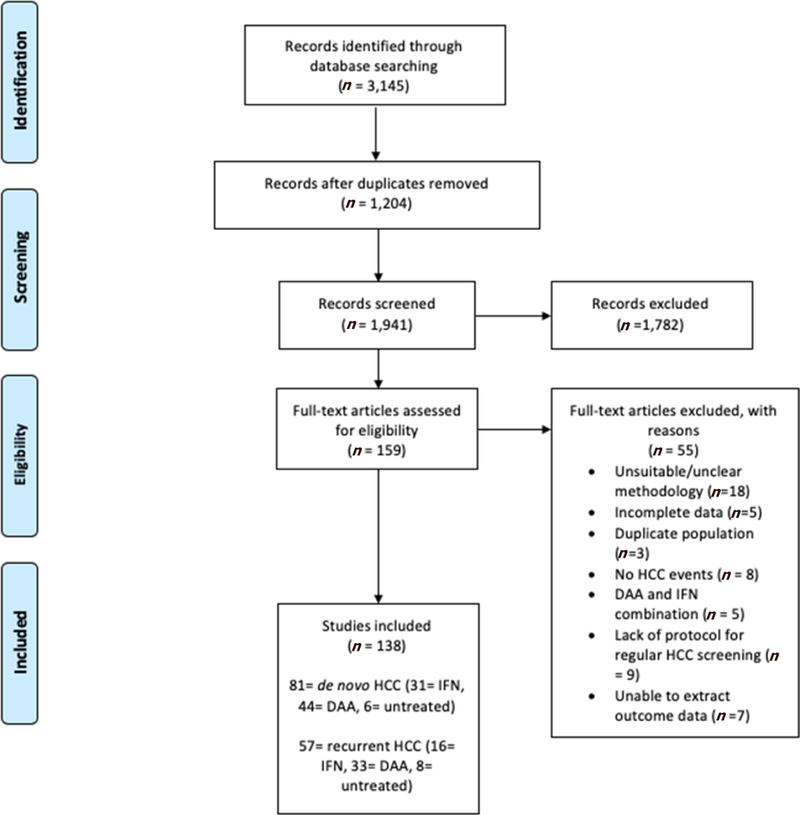
PRISMA study flow chart. HCC: hepatocellular carcinoma.

We retrieved 3145 citations after the electronic database search. After excluding duplicates and studies which did not pertain to the patient population or outcomes in question, we included a total of 138 studies (*n* = 177,512). We found 81 studies looking at *de novo* HCC occurrence (*n* = 172,636): 31 studies of IFN-treated persons (*n* = 71,443), 44 studies of DAA-treated patients (*n* = 91,249) and 6 relating to untreated subjects (*n* = 9944). We included 57 studies which evaluated HCC recurrence (*n* = 4876): 16 studies of IFN-treated populations (*n* = 1043), 33 relating to DAA-treated (*n* = 2186) and 8 studies of untreated patients (*n* = 1647). There were 16 DAA studies which examined both *de novo* and recurrence rates of HCC. Figure 1 contains study flow chart. Supplementary Tables 3 and 4 contain individual study details.

Both groups had similarly high rates of male patients, due to the inclusion of large studies of Veterans Affairs (VA) hospitals: 91% in the DAA group and 90.3% in the IFN group (*P* = 0.1992). The rate of SVR in the DAA-treated group was 88.9%, compared to 45.9% in the IFN-treated group (*P* ≤ 0.001). Overall, mean age was higher in the DAA-treated *vs.* IFN-treated group (58.4 *vs.* 52.6 years; *P* = 0.0073), as was the prevalence of diabetes (34.5% *vs.* 11.7%; *P* ≤ 0.001). As expected, mean follow-up was longer in the IFN group: 7.75 *vs.* 1.46 years (*P* ≤ 0.001). DAA-treated patients had higher prevalent cirrhosis compared to IFN-treated patients (47.8% *vs.* 34.2%, *P* = 0.0017), and among persons with cirrhosis, Child-Pugh stage B/C disease was more frequent in the DAA-treated group (19.4% *vs.* 3.0%, *P* ≤ 0.001). The AFP levels at the time of initiation of HCV treatment were similar in both groups (6.2 ng/mL in DAA *vs.* 5.6 ng/mL in IFN-treated, (*P* = 0.4456)). Mean platelet count was lower in the DAA group (155 × 10^9^/L *vs.* 197 × 10^9^/L), (*P* ≤ 0.001), as was mean albumin (3.8 g/dL *vs.* 4.1 g/dL in IFN (*P* ≤ 0.001). The prevalence of genotype 1 (GT-1) was 60.8% in the IFN and 85.0% in DAA group (*P* = 0.0016); the prevalence of GT-3 was not significantly different between groups: 5.1% in DAA *vs.* 12.4% in IFN (*P* = 0.8381) [see Table 1].

### Rates of *de novo* HCC

**Figure 2.**
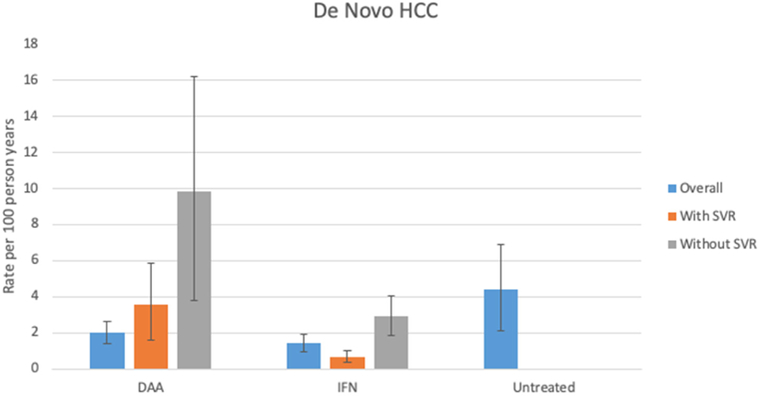
Rates of *de novo* HCC by treatment group and SVR status. Colored bars represent rates of *de novo* HCC by SVR status, with 95%CIs depicted by the capped 95%CI vertical black lines. Rates of *de novo* HCC were as follows: DAA group, overall: 2.01/100py (95%CI: 1.38, 2.67), SVR: 3.57/100py (95%CI: 1.63, 5.88) and non-SVR: 9.83/100py (: 3.77,16.22). IFN group, overall: 1.45/100py (95%CI: 0.98, 1.94), SVR: 0.70/100py, (95%CI: 0.41, 1.04), non-SVR: 2.94/100py (95%CI: 1.88, 4.04). Untreated group: 4.41/100py (95%CI: 2.10, 6.90). SVR: sustained viral response; IFN: interferon; DAA: direct-acting antiviral; CI: confidence interval; HCC: hepatocellular carcinoma

The estimated incidence of *de novo* HCC occurrence after DAA treatment was calculated to be 2.01/100py (95%CI: 1.38, 2.67) compared to 1.45/100py (95%CI: 0.98, 1.94) in IFN-treated subjects. In patients who had never been treated with HCV therapy, the rate of *de novo* HCC was significantly higher than IFN-treated groups at 4.41/100py (95%CI: 2.10, 6.90). We performed a sub-group analysis by SVR status: patients treated with DAAs who achieved SVR developed HCC at a rate of 3.57 per 100py (95%CI: 1.63, 5.88) while the IFN-treated SVR group had a lower estimated incidence rate of 0.70/100py, (95%CI: 0.41, 1.04). The DAA SVR sub-group had an unexpectedly higher rate of HCC occurrence (although not statistically significant) than the overall DAA group. This may be explained by the fact that not all studies reported SVR status, so marginally smaller numbers were available for this sub-group analysis, which may have arbitrarily included the studies with higher rates (*n* = 87,952 in the sub-group analysis compared to 91,249 in the entire DAA group). In the non-SVR sub-groups, DAA-treated patients developed HCC at a rate of 9.83/100py (95%CI: 3.77,16.22). Their IFN-treated counterparts who did not clear HCV had a *de novo* HCC incidence rate of 2.94/100py (95%CI: 1.88, 4.04) [Figure 2].

**Figure 3.**
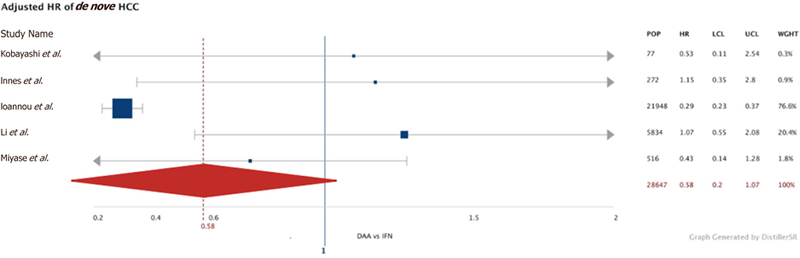
Adjusted hazards ratio of risk of *de novo* HCC in DAA- *vs.* IFN-treated populations. The multivariate-adjusted hazard ratio of each individual study is represented by the blue square with the size of the square being proportional to the n of the study. The thin horizontal grey bars represent the 95%CI of each study and the thick vertical blue line marks where the HR is equal to 1. The red diamond with the dashed vertical red line is the overall adjusted HR, which was 0.58 (95%CI: 0.20, 1.07) for *de novo* HCC in the DAA population compared to the IFN-treated population. HCC: hepatocellular carcinoma; IFN: interferon; CI: confidence interval

We performed an additional smaller meta-analysis of the five studies that performed multivariate-adjusted hazard ratios (adjusting for gender, baseline cirrhosis, and patient age), and found no increased risk of *de novo* HCC in patients treated with DAA compared to the IFN-treated population: unadjusted HR of 1.76 (95%CI: 0.001, 4.70) and adjusted HR of 0.58 (95%CI: 0.20, 1.07) [Figure 3]. When we looked at *de novo* rates in the second year after treatment with DAAs, we found a similar incidence rate of 0.88 per 100py, (95%CI: 0.0001, 1.94) compared to a second-year annualized incidence rate of 0.55 per 100py, (95%CI: 0.03, 1.29) in IFN-treated patients. We then excluded any cases of *de novo* HCC occurring within six months after end-of-treatment and obtained an incidence rate of 1.12 per 100py (95%CI: 0.43,1.98) in the DAA group and a higher incidence rate of 3.01 per 100py (95%CI: 0.033, 9.02) in the IFN group, which did not reach statistical significance.

### Rates of recurrent HCC

**Figure 4.**
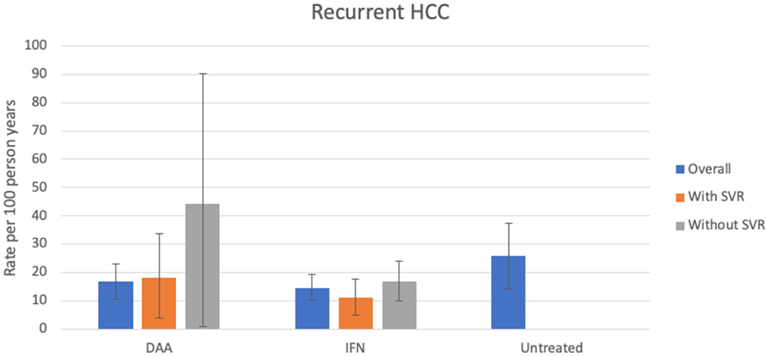
Rates of recurrent HCC by treatment group and SVR status. Colored bars represent rates of recurrent HCC by SVR status, with 95%CIs depicted by the capped vertical black lines. Rates of recurrent HCC were as follows: DAA group, overall: 16.76/100py (95%CI: 10.75, 22.91), SVR: 18.17/100py (95%CI: 3.84, 33.58) and non-SVR: 44.16/100py, (95%CI: 0.006, 90.35). IFN group, overall: 14.31/100py (95%CI: 10.17, 19.16), SVR: 11.01/100py (95%CI: 4.85, 17.63), non-SVR: 16.89/100py (95%CI: 10.05, 24.02). Untreated group: 25.69/100py (95% CI: 14.44,37.27). “Overall” group included some patients not included in SVR or non-SVR groups. SVR: sustained viral response; HCC: HCC: hepatocellular carcinoma; CI: confidence interval; IFN: interferon; DAA: direct-acting antiviral

The recurrence rate of HCC was relatively high across all groups: 16.76/100py (95%CI: 10.75, 22.91) and 14.31/100py (95%CI: 10.17, 19.16) in DAA- and IFN-treated populations respectively. It was similar between untreated patients and IFN-treated patients without SVR [25.69/100py (95%CI: 14.44,37.27) compared to 16.89/100py (95%CI: 10.05, 24.02)], suggesting that IFN does not have significant anti-tumor effect in the absence of viral clearance. Patients treated with DAA who did not achieve SVR appeared to have a high rate of recurrent HCC; however, the numbers of patients with DAA failure were small, thus the CI is wide: 44.16/100py, (95%CI = 0.006, 90.35). DAA-treated patients who achieved SVR had a recurrence rate of 18.17/100py (95%CI: 3.84, 33.58) and in IFN-treated populations with SVR the recurrence rate was not significantly different at 11.01/100py (95%CI: 4.85, 17.63) [Figure 4].

**Figure 5.**
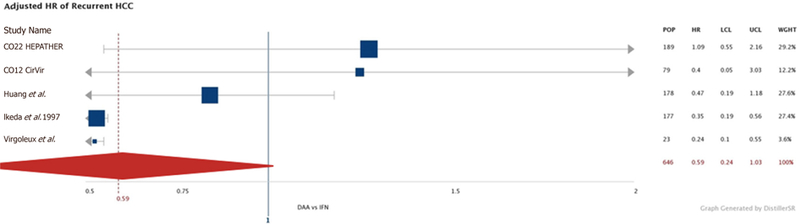
Adjusted hazards ratio of risk of recurrent HCC in DAA- vs. IFN-treated populations. The multivariate-adjusted hazard ratio of each individual study is represented by the blue square with the size of the square being proportional to the n of the study. The thin horizontal grey bars represent the 95%CI of each study and the thick vertical blue line marks where the HR is equal to 1. The red diamond with the dashed vertical red line is the overall adjusted HR, which was 0.59 (95%CI: 0.24, 1.03) for recurrent HCC in the DAA population compared to the IFN-treated population. IFN: interferon; DAA: direct-acting antiviral; HCC: hepatocellular carcinoma

When HCC recurrence that occurred during the first six months post-completion of treatment was excluded, the rates were similar between DAA and IFN groups: 10.75/100py, (95%CI: 5.50, 16.30) *vs.* 14.62/100py, (95%CI: 8.94, 20.52). Again, when we take the second-year rates post-treatment, we saw similar recurrence rates [DAA group: 6.66/100py (95%CI: 1.96, 12.11) and IFN group: 5.35/100py, (95%CI: 0.54, 11.06)]. Our additional meta-analysis of the five studies with multivariate analyses found that the unadjusted HR of recurrence in DAA *vs.* IFN-treated groups was 0.59 (95%CI: 0.11, 1.14) and after adjusting for age, gender, baseline AFP, ethnicity and Child-Pugh score, the HR was 0.59 (95%CI: 0.24, 1.03) [Figure 5].

## DISCUSSION

Our study is the largest meta-analysis to evaluate the risk of HCC after treatment with DAA therapy published to date. The results demonstrate that the risk of *de novo* HCC is similar between IFN- and DAA-treated cohorts. In the sub-group analysis by SVR, non-SVR IFN and non-SVR DAA groups had similar rates of *de novo* HCC, although the confidence interval for the DAA cohort was wide because of the very small numbers who did not achieve SVR. Those who achieved SVR with IFN had a significantly lower rate of HCC occurrence than the DAA-treated SVR group. We postulate that this is because patients who could tolerate and achieve viral clearance with IFN therapy were a very well-compensated group with minimal liver disease. Indeed, we observed that the entire IFN-treated group was significantly younger and had less cirrhosis, less diabetes and lower Child-Pugh scores than DAA-treated patients. This echoes many of the other previously published studies^[20–23]^. In our meta-analysis of hazard ratios, after adjusting for a number of risk factors for HCC, we found that the rates of *de novo* HCC were lower in the DAA-treatment group compared to IFN-treated, although this did not reach statistical significance. This finding reinforces the hypothesis that “higher-risk” patients receive treatment with DAA agents than were treated with IFN in the past, thus leading to selection bias. There has been particular concern about the rates of HCC recurrence following DAA treatment, which were felt to be even more pronounced than the risk of *de novo* HCC^[12,14,24]^. However, we found no increased risk of HCC in DAA-treated patients compared with IFN-treated patients, and after adjusting for risk factors such as age and cirrhosis, the DAA-treated group trended towards a lower rate of recurrent HCC, although this did not reach statistical significance.

Our meta-analysis excluded any studies with less than one year of follow-up after end-of-treatment; this rigorous exclusion was not performed in another recent meta-analysis^[25]^. We believe that helped to mitigate any increase in rates of “early” HCC post-treatment due to sub-clinical HCC which may have been present prior to the initiation of antiviral therapy. We also performed a sub-group analysis, whereby HCC events occurring within six months of end-of-treatment were excluded, and we measured HCC rates in the second-year post-treatment. Our strict exclusion criteria and subgroup analyses were designed to mitigate surveillance bias, where patients who undergo treatment for HCV may be monitored more closely in the months following treatment due to more frequent visits to a hepatologist and may be more likely to undergo HCC screening with abdominal imaging. Limitations of our study include its retrospective observational nature thus allowing for confounding variables since many of these studies were not initially designed to compare rates of HCC, and the heterogeneous nature of the studies which had variable lengths of follow-up, differing percentages of patients with cirrhosis and different individual DAA treatment regimens. Furthermore, none of the studies which included both DAA-treated and IFN-treated persons adjusted for the exact same baseline risk factors for HCC, so this limited the validity of comparing the studies directly and deriving hazard ratios. There was minimal accounting for indication bias, which is one of the criticisms of these studies. Finally, there was a disproportionate number of male patients included in the meta-analysis, due to the high number of subjects in the VA studies: 64,306/93,435 DAA-treated and 50,143/72,486 IFN-treated subjects included in the meta-analysis were acquired from VA-based studies. Given that the veteran patient population has higher rates of smoking and alcohol use than the general population, the risk of HCC in this subgroup was likely higher which may have skewed the results (although approximately equal proportions of DAA and IFN-treated patients were obtained from VA data).

The debate on whether or not DAAs increase the risk of HCC has been ongoing for several years now. Initial reports from Europe first raised concern, and multiple studies confirming and refuting this theory have since been published ^[12–18,26]^. The immune theory and liver regeneration theory are some of the most commonly cited theories for the perceived increase in HCC after treatment with DAA therapy. After treatment with DAAs, the HCV virus becomes undetectable within days to weeks, far more quickly than with IFN-based therapy. It has been suggested that clearing the hepatitis virus rapidly with fall in antigenic load removes the immune surveillance (with CD8+ T cells for example) which protected against the development of neoplasia^[27]^. It is also thought that as the liver regenerates rapidly after viral clearance, small sub-clinical tumors or areas of metaplasia may grow and become clinically evident^[28]^.

Small case series have found that tumors are more likely to be multi-focal and tend to have a more aggressive biology in DAA-treated individuals compared to IFN-treated or untreated subjects. Romano *et al*.^[29]^ describe a particularly aggressive HCC pattern at diagnosis after DAA treatment. In 39% of the 27 patients treated with DAA therapy who developed HCC, there was an infiltrative pattern or more than three nodules present (25% of these cases had vascular invasion or extrahepatic spread). Renzulli *et al*.^[30]^ found imaging features of microvascular invasion in 70.7% of HCC nodules after DAA treatment; microvascular invasion was present in only 33.3% of HCC nodules that occurred before DAA treatment. However, conflicting studies have also been published: the large CIR-VIR study found infiltrative HCC in 10.8%^[26]^; typically up to 13% of all HCC cases are found to be infiltrating and are often associated with background hepatitis B infection^[31]^. The study by Zanetto *et al*.^[32]^ evaluated 9 DAA-treated explanted livers with 14 control (untreated) liver explants and found no difference in median number and total tumor volume of HCC nodules, tumor differentiation, or microvascular invasion. Clearly, more studies evaluating the biology of tumors after DAA treatment are required before a definitive conclusion can be drawn.

The high efficacy and tolerability of DAAs has resulted in their use in patients who have more intrinsic risk factors for HCC, including advanced age, diabetes and cirrhosis. Several studies have shown the patients treated with DAAs have more risk factors than historical IFN-treated cohorts. The US Veterans Administration study by Li *et al*.^[20]^ demonstrated the “warehousing” of HCV-infected patients which took place in the years leading up to the release of DAAs. They showed that patients who received the first available DAA agents had the most advanced liver disease and higher rates of HCC as a result, because HCV treatment had been deferred in anticipation of an efficacious tolerable regimen.

Fangazio *et al*.^[33]^ showed that patients who developed *de novo* or recurrent HCC after DAA treatment were less likely to achieve SVR (SVR12 rate of 64% in patients with HCC compared to 95% in their counterparts without HCC, which is much more typical of the DAA viral clearance rates). This finding suggests that in patients who do not achieve SVR with DAA, clinicians should have heightened levels of suspicion for underlying undetected HCC. A study by Beste *et al*.^[34]^ also found that HCC patients had lower rates of viral clearance than patients without HCC, even after adjusting for cirrhosis and genotype. It has been postulated that the virus within tumor cells could be inaccessible to DAAs because of differential blood supply, which prevents the clearance of virus. Furthermore, HCC arises in the setting of chronic inflammation with alterations in the hepatic architecture and micro-environment, including cytokine and chemokine populations^[35]^. This altered immune environment may predispose to treatment failure and to the development of liver cancer. A study by Tachi *et al*.^[36]^ revealed that higher total bilirubin levels and higher liver stiffness measurements (as measured by ARFI elastography) prior to DAA treatment were positively associated with occurrence of HCC after achievement of SVR with DAA therapy. Clearly a risk of HCC still exists even after SVR with DAA treatment, so surveillance imaging should not be ceased even after treatment success.

Even in view of the mixed data, it is evident that the achievement of SVR is the ultimate arbiter of risk of HCC. While many studies have shown no increased risk of HCC after DAA treatment, multiple studies have demonstrated a lower risk of HCC in DAA-treated patients who achieve SVR compared to untreated patients^[22,23,37]^. Treatment with DAAs also portends other benefits such as a decrease in MELD and Child-Pugh score (which sometimes results in delisting for liver transplant and the so-called MELD “purgatory”), and a reduction in the risk of death as demonstrated by the French Hepather cohort^[23,38–46]^. Munoz *et al*.^[39]^ have estimated that the DAA-induced reduction in MELD score to below the threshold for liver transplantation listing may occur in 592–993 listed patients/year during the first year after treatment, and that 213–515 donated livers/year could be redistributed as a result. As more time passes since their development and additional studies with longer follow-up are published, the benefit of treatment with DAAs and the lack of a causative effect on carcinogenesis becomes clearer. It is now evident that withholding DAA treatment denies patients the possibility of a significant improvement in liver disease and consigns patients to a higher risk of HCC development.

In conclusion, we did not find evidence of increased rates of *de novo* HCC or recurrence in DAA-treated compared with IFN-treated patients. Compared to those treated with IFN, older patients with additional pre-existing risk factors for HCC development were treated with DAA. This imbalance would appear to explain the higher numerical incidence of *de novo* HCC among DAA-treated patients. Given the success and cost-effectiveness of DAA therapy for the treatment of HCV infection^[47–49]^, clinicians should not be dissuaded by prior studies that suggest an increased risk of precipitating HCC development, as this seems to largely be a product of the presence of more advanced liver disease and increased risk factors among DAA-treated patients. Rather, the practice of continued surveillance for HCC for those persons with baseline risk factors, should continue to be reinforced.

## Supplementary Material

Supplemental table1

Supplemental table2

Supplemental table3

Supplemental table4

## Figures and Tables

**Table 1.** Baseline characteristics in the DAA-treated and IFN-treated groups

Characteristic	DAA group	IFN group	*P* value
Sex, % male	91.0	90.3	0.1992
Age in years, mean ± SD	58.4 ± 6.05	52.6 ± 7.21	0.0073
Follow-up in years, mean ± SD	1.46 ± 0.54	7.75 ± 3.23	< 0.001
Cirrhosis, %	47.8	34.2	0.0017
Child-Pugh B/C, %	19.4	3.0	< 0.001
AFP level, ng/mL, mean ± SD	6.2 ± 5.4	5.6 ± 4.3	0.4456
Platelet count, mean ± SD	155 × 10^9^/L ± 30	197 × 10^9^/L ± 28	< 0.001
Albumin in g/dL, mean ± SD	3.8 ± 0.3	4.1 ± 0.3	< 0.001
Prevalence of genotype 1, %	85.0	60.8	0.0016
Prevalence of genotype 3, %	5.1	12.4	0.8381

AFP: alpha fetoprotein; DAA: direct-acting antiviral; IFN: interferon; SD: standard deviation

## References

[1] Mittal S, El-Serag HB. Epidemiology of hepatocellular carcinoma: consider the population. J Clin Gastroenterol 2013;47:S2–6. 23632345 10.1097/MCG.0b013e3182872f29 PMC3683119

[2] Lemon SM, McGivern DR. Is hepatitis C virus carcinogenic? Gastroenterology 2012;142:1274–8. 22537433 10.1053/j.gastro.2012.01.045 PMC4422399

[3] Bruix J, Sherman M. Management of hepatocellular carcinoma: an update. Hepatology 2011;53:1020–2. 21374666 10.1002/hep.24199 PMC3084991

[4] Hassan MM, Hwang LY, Hatten CJ, Swaim M, Li D, Risk factors for hepatocellular carcinoma: synergism of alcohol with viral hepatitis and diabetes mellitus. Hepatology 2002;36:1206–13. 12395331 10.1053/jhep.2002.36780

[5] Sanyal AJ, Yoon SK, Lencioni R. The Etiology of Hepatocellular Carcinoma and Consequences for Treatment. Oncologist 2010;15:14–22. 21115577 10.1634/theoncologist.2010-S4-14

[6] Janjua NZ, Chong M, Kuo M, Woods R, Wong J, Long-term effect of sustained virological response on hepatocellular carcinoma in patients with hepatitis C in Canada. J Hepatology 2017;66:504–13. 10.1016/j.jhep.2016.10.028 27818234

[7] Brown JL. Interferon therapy reduces the risk for hepatocellular carcinoma. Gut 2000;47:610–1. 11034573 10.1136/gut.47.5.610 PMC1728124

[8] Ikeda M, Fujiyama S, Tanaka M, Sata M, Ide T, Risk factors for development of hepatocellular carcinoma in patients with chronic hepatitis C after sustained response to interferon. J Gastroenterol 2005;40:148–56. 15770398 10.1007/s00535-004-1519-2

[9] Singal AK, Singh A, Jaganmohan S, Guturu P, Mummadi R, Antiviral Therapy Reduces Risk of Hepatocellular Carcinoma in Patients With Hepatitis C Virus-Related Cirrhosis. Clin Gastroenterol H 2010;8:192–9. 10.1016/j.cgh.2009.10.026 19879972

[10] Fried MW, Shiffman ML, Reddy KR, Smith C, Marinos G, Peginterferon alfa-2a plus ribavirin for chronic hepatitis C virus infection. N Engl J Med 2002;347:975–82. 12324553 10.1056/NEJMoa020047

[11] Harris RJ, Thomas B, Griffiths J, Costella A, Chapman R, Increased uptake and new therapies are needed to avert rising hepatitis C-related end stage liver disease in England: Modelling the predicted impact of treatment under different scenarios. J Hepatol 2014;61:530–7. 24824282 10.1016/j.jhep.2014.05.008

[12] Reig M, Mariño Z, Perelló C, Iñarrairaegui M, Ribeiro A, Unexpected high rate of early tumor recurrence in patients with HCV-related HCC undergoing interferon-free therapy. J Hepatol 2016;65:719–26. 27084592 10.1016/j.jhep.2016.04.008

[13] Kozbial K, Moser S, Schwarzer R, Laferl H, Al-Zoairy R, Unexpected high incidence of hepatocellular carcinoma in cirrhotic patients with sustained virologic response following interferon-free direct-acting antiviral treatment. J Hepatol 2016;65:856–8. 27318327 10.1016/j.jhep.2016.06.009

[14] Conti F, Buonfiglioli F, Scuteri A, Crespi C, Bolondi L, Early occurrence and recurrence of hepatocellular carcinoma in HCV-related cirrhosis treated with direct-acting antivirals. J Hepatol 2016;65:727–33. 27349488 10.1016/j.jhep.2016.06.015

[15] Cardoso H, Vale AM, Rodrigues S, Gonçalves R, Albuquerque A, High incidence of hepatocellular carcinoma following successful interferon-free antiviral therapy for hepatitis C associated cirrhosis. J Hepatol 2016;65:1070–1. 27476768 10.1016/j.jhep.2016.07.027

[16] Cheung MC, Walker AJ, Hudson BE, Verma S, McLauchlan J, Outcomes after successful direct-acting antiviral therapy for patients with chronic hepatitis C and decompensated cirrhosis. J Hepatol 2016;65:741–7. 27388925 10.1016/j.jhep.2016.06.019

[17] Zeng QL, Li ZQ, Liang HX, Xu GH, Li CX, Unexpected high incidence of hepatocellular carcinoma in patients with hepatitis C in the era of DAAs: Too alarming? J Hepatol 2016;65:1068–9. 27476763 10.1016/j.jhep.2016.07.029

[18] Zavaglia C, Okolicsanyi S, Cesarini L, Mazzarelli C, Pontecorvi V, Is the risk of neoplastic recurrence increased after prescribing direct-acting antivirals for HCV patients whose HCC was previously cured? J Hepatol 2017;66:236–7. 27592303 10.1016/j.jhep.2016.08.016

[19] Nault JC, Colombo M. Hepatocellular carcinoma and direct acting antiviral treatments: controversy after the revolution. J Hepatol 2016;65:663–5. 27417216 10.1016/j.jhep.2016.07.004

[20] Li DK, Ren Y, Fierer DS, Rutledge S, Shaikh OS, The short-term incidence of hepatocellular carcinoma is not increased after hepatitis C treatment with direct-acting antivirals: An ERCHIVES study. Hepatology 2018;67:2244–53. 29205416 10.1002/hep.29707

[21] Kobayashi M, Suzuki F, Fujiyama S, Kawamura Y, Sezaki H, Sustained virologic response by direct antiviral agents reduces the incidence of hepatocellular carcinoma in patients with HCV infection. J Med Virol 2017;89:476–83. 27531586 10.1002/jmv.24663

[22] Kanwal F, Kramer J, Asch SM, Chayanupatkul M, Cao Y, Risk of hepatocellular cancer in HCV patients treated with direct-acting antiviral agents. Gastroenterology 2017;153:996–1005. 28642197 10.1053/j.gastro.2017.06.012

[23] Carrat F Clinical outcomes in HCV-infected patients treated with direct acting antivirals-18 month post-treatment follow-up in the french anrs CO22 hepather cohort study. J Hepatol 2016;64:S215.

[24] El Kassas M, Funk AL, Salaheldin M, Shimakawa Y, Eltabbakh M, Increased recurrence rates of hepatocellular carcinoma after DAA therapy in a hepatitis C infected Egyptian cohort: a comparative analysis. J Viral Hepat 2018;25:623–30. 29274197 10.1111/jvh.12854

[25] Waziry R, Hajarizadeh B, Grebely J, Amin J, Law M, Hepatocellular carcinoma risk following direct-acting antiviral HCV therapy: A systematic review, meta-analyses, and meta-regression. J Hepatol 2017;67:1204–12. 28802876 10.1016/j.jhep.2017.07.025

[26] Pol S Lack of evidence of an effect of Direct Acting Antivirals on the recurrence of hepatocellular carcinoma. J Hepatol 2016;65:734–40. 27288051 10.1016/j.jhep.2016.05.045

[27] Butt AS, Sharif F, Abid S. Impact of direct acting antivirals on occurrence and recurrence of hepatocellular carcinoma: Biologically plausible or an epiphenomenon? World J Hepatol 2018;10:267–76. 29527262 10.4254/wjh.v10.i2.267 PMC5838445

[28] Grandhe S, Frenette CT. Occurrence and Recurrence of Hepatocellular Carcinoma After Successful Direct-Acting Antiviral Therapy for Patients With Chronic Hepatitis C Virus Infection. Gastro Hepat 2017;13:421. PMC5572972 28867970

[29] Romano A, Capra F, Piovesan S, Chemello L, Cavalletto L, Incidence and pattern of” de novo” hepatocellular carcinoma in HCV patients treated with oral DAAs. In. Hepatology: Wiley-blackwell 111 river st, hoboken 07030–5774, NJ USA; 2016.

[30] Renzulli M, Buonfiglioli F, Conti F, Brocchi S, Serio I, Imaging features of microvascular invasion in hepatocellular carcinoma developed after direct-acting antiviral therapy in HCV-related cirrhosis. Eur Radiol 2018;28:506–13. 28894901 10.1007/s00330-017-5033-3

[31] Demirjian A, Peng P, Geschwind JFH, Cosgrove D, Schutz J, Infiltrating hepatocellular carcinoma: seeing the tree through the forest. J Gastrointest Surg 2011;15:2089–97. 21725699 10.1007/s11605-011-1614-7 PMC3580771

[32] Zanetto A, Shalaby S, Vitale A, Mescoli C, Ferrarese A, Drop-out rate from the liver transplant waiting list due to HCC progression in HCV-infected patients treated with direct acting antivirals. Liver Transpl 2017;23:1103–12. 28544587 10.1002/lt.24790

[33] Fangazio S, Camatta D, Minh MT, Ceriani E, Minisini R, Treatment failure after interferon-free treatment of hepatitis C as a clue of a yet undetected hepatocellular carcinoma. J Hepatol 2018;68:212–3. 10.1016/j.jhep.2017.06.037 28890357

[34] Beste LA, Green PK, Berry K, Kogut MJ, Allison SK, Effectiveness of hepatitis C antiviral treatment in a USA cohort of veteran patients with hepatocellular carcinoma. J Hepatol 2017;67:32–9. 28267622 10.1016/j.jhep.2017.02.027 PMC6590903

[35] Hengst J, Schlaphoff V, Deterding K, Falk C, Manns M, DAA-induced HCV clearance does not restore the altered cytokine and chemokine milieu in patients with chronic hepatitis C. J Hepatol 2016;64:S417–S8. 10.1093/infdis/jiw457 27683821

[36] Tachi Y, Hirai T, Kojima Y, Ishizu Y, Honda T, Liver stiffness measurement predicts hepatocellular carcinoma development in patients treated with direct-acting antivirals. JGH Open 2017;1:44–9. 30483532 10.1002/jgh3.12007 PMC6207000

[37] Adhoute X, Castellani P, Bourlière M. Impact of direct-acting antiviral agents on the risk for hepatocellular carcinoma. Transl Gastroenterol Hepatol 2017;2:110. 29354767 10.21037/tgh.2017.12.04 PMC5762991

[38] Belli LS, Berenguer M, Cortesi PA, Strazzabosco M, Rockenschaub SR, Delisting of liver transplant candidates with chronic hepatitis C after viral eradication: A European study. J Hepatol 2016;65:524–31. 27212241 10.1016/j.jhep.2016.05.010

[39] Munoz SJ, Reich DJ, Rothstein KD, Xiao GS, Patel V, Curing decompensated wait listed HCV patients with the new DAAs: the potential significant impact on liver transplant wait list and organ allocation. Hepatology 2015;62:311A.

[40] Manns M, Samuel D, Gane EJ, Mutimer D, McCaughan G, Ledipasvir and sofosbuvir plus ribavirin in patients with genotype 1 or 4 hepatitis C virus infection and advanced liver disease: a multicentre, open-label, randomised, phase 2 trial. Lancet Infect Dis 2016;16:685–97. 26907736 10.1016/S1473-3099(16)00052-9

[41] Poordad F, Schiff ER, Vierling JM, Landis C, Fontana RJ, Daclatasvir with sofosbuvir and ribavirin for hepatitis C virus infection with advanced cirrhosis or post-liver transplantation recurrence. Hepatology 2016;63:1493–505. 26754432 10.1002/hep.28446 PMC5069651

[42] Jacobson I, Poordad F, Firpi-Morell R, Everson G, Verna E, O008: Efficacy and safety of grazoprevir and elbasvir in hepatitis C genotype 1-infected patients with child-pugh class B cirrhosis (C-salt part A). J Hepatol 2015;62:S193–S4.

[43] Curry MP, O’Leary JG, Bzowej N, Muir AJ, Korenblat KM, Sofosbuvir and velpatasvir for HCV in patients with decompensated cirrhosis. N Engl J Med 2015;373:2618–28. 26569658 10.1056/NEJMoa1512614

[44] Aqel BA, Pungpapong S, Leise M, Werner K, Chervenak AE, Multicenter experience using simeprevir and sofosbuvir with or without ribavirin to treat hepatitis C genotype 1 in patients with cirrhosis. Hepatology 2015;62:1004–12. 26096332 10.1002/hep.27937

[45] Deuffic-Burban S, Mathurin P, Rosa I, Bouvier AM, Cannesson A, Impact of emerging hepatitis C virus treatments on future needs for liver transplantation in France: a modelling approach. Digest Liver Dis 2014;46:157–63. 10.1016/j.dld.2013.08.137 24119483

[46] Ponziani FR, Mangiola F, Binda C, Zocco MA, Siciliano M, Future of liver disease in the era of direct acting antivirals for the treatment of hepatitis C. World J Hepatol 2017;9:352–67. 28321272 10.4254/wjh.v9.i7.352 PMC5340991

[47] Chan K, Lai MN, Groessl EJ, Hanchate AD, Wong JB, Cost effectiveness of direct-acting antiviral therapy for treatment-naive patients with chronic HCV genotype 1 infection in the veterans health administration. Clin Gastroenterol Hepatol 2013;11:1503–10. 23707354 10.1016/j.cgh.2013.05.014

[48] He T, Lopez-Olivo M, Hur C, Chhatwal J. Systematic review: cost-effectiveness of direct-acting antivirals for treatment of hepatitis C genotypes 2–6. Aliment Pharmacol Ther 2017;46:711–21. 28836278 10.1111/apt.14271

[49] Chhatwal J, He T, Lopez-Olivo MA. Systematic review of modelling approaches for the cost effectiveness of hepatitis C treatment with direct-acting antivirals. Pharmacoeconomics 2016;34:551–67. 26748919 10.1007/s40273-015-0373-9

