# Supplementary material for: No evidence for higher rates of hepatocellular carcinoma after direct-acting antiviral treatment: a meta-analysis
Source: Hepatoma Res. Author manuscript; Available in PMC 2019 Aug 26. (PMC6709867; doi:10.20517/2394-5079.2019.19)
Supplement: Supplemental table1 [file NIHMS1046854-supplement-Supplemental_table1.docx]

| **Study** | **Ascertainment of cirrhosis** | **Time since last imaging*** | **Ascertainment of SVR** | **Control group/ comparator** | **Adjusted for potential confounders** | **Follow-up duration#** | **Assessment during f/u** | **Assessment of HCC event** | **Time interval between HCC and initiation of HCV therapy^** | **Data on initial HCC management** |
| --- | --- | --- | --- | --- | --- | --- | --- | --- | --- | --- |
| **DAA recurence studies** |  |  |  |  |  |  |  |  |  |  |
| **Yang, 2016** |  | **?** | **+** | **+** | **-** | **?** | **+** | **+** | **?** | **+** |
| **Pol, CO22,2016** |  | **?** | **+** | **+** | **+** | **+** | **?** | **?** | **+** | **?** |
| **Pol, CO12, 2016** |  | **?** | **+** | **+** | **+** | **+** | **+** | **+** | **-** | **+** |
| **Pol, CO23, 2016** |  | **?** | **+** | **-** | **+** | **+** | **?** | **+** | **+** | **+** |
| **Zavaglia, 2017** |  | **+** | **+** | **-** | **+** | **+** | **?** | **?** | **+** | **+** |
| **Torres, 2016** |  | **?** | **+** | **-** | **+** | **+** | **?** | **?** | **+** | **+** |
| **Adhoute, 2018** |  | **+** | **+** | **+** | **+** | **+** | **+** | **+** | **+** | **+** |
| **Petta, 2017** |  | **+** | **+** | **+** | **-** | **+** | **+** | **+** | **-** | **+** |
| **Minami, 2016** |  | **?** | **+** | **+** | **+** | **+** | **?** | **?** | **+** | **+** |
| **Reig, 2017** |  | **+** | **+** | **+** | **+** | **-** | **+** | **+** | **+** | **+** |
| **Huang, 2017** |  | **?** | **+** | **+** | **+** | **?** | **+** | **+** | **?** | **+** |
| **Ikeda, 2017** |  | **+** | **+** | **+** | **+** | **+** | **+** | **+** | **+** | **+** |
| **Cabibbo, RESIST, 2017** |  | **+** | **+** | **+** | **+** | **-** | **+** | **+** | **+** | **+** |
| **Kolly, 2017** |  | **+** | **+** | **-** | **+** | **+** | **+** | **+** | **+** |  |
| **Virlogeuz, 2017** |  | **+** | **+** | **+** | **+** | **+** | **+** | **+** | **+** | **+** |
| **Sangiovanni, 2017** |  | **+** | **+** | **-** | **+** | **+** | **+** | **?** | **+** | **-** |
| **Minami, 2017** |  |  | **+** | **-** | **+** | **+** | **+** | **+** | **+** | **+** |
| **Urabe, 2018** |  |  | **-** | **+** | **+** | **-** | **+** | **+** | **+** | **-** |
| **Lleo, 2018** |  |  | **+** | **-** | **+** | **+** | **+** | **+** | **+** | **+** |
| **DAA occurrence studies** |  |  |  |  |  |  |  |  |  |  |
| **Romano, 2018** | **+** | **+** | **+** | **-** | **+** | **+** | **+** | **+** |  |  |
| **Kobayashi, 2017** | **+** | **-** | **+** | **+** | **+** | **+** | **+** | **+** |  |  |
| **Cardoso, 2016** | **+** | **?** | **+** | **?** | **+** | **+** | **?** | **?** |  |  |
| **Toyoda, 2016** | **+** | **-** | **+** | **+** | **+** | **+** | **?** | **?** |  |  |
| **Mangia, 2016** | **-** | **+** | **+** | **-** | **+** | **+** | **+** | **+** |  |  |
| **Innes, 2018** | **-** | **?** | **+** | **+** | **+** | **+** | **-** | **-** |  |  |
| **Ji, 2017** | **?** | **?** | **+** | **+** | **+** | **+** | **?** | **?** |  |  |
| **Korenaga, 2018** | **-** | **+** | **+** | **+** | **+** | **+** | **+** | **+** |  |  |
| **Calvaruso, 2018** | **+** | **+** | **+** | **-** | **+** | **+** | **+** | **+** |  |  |
| **Ravi, 2017** | **?** | **+** | **+** | **-** | **+** | **-** | **+** | **+** |  |  |
| **Muir, 2018** | **+** | **+** | **+** | **-** | **+** | **+** | **+** | **+** |  |  |
| **Sogni, CO13, 2016** | **+** | **?** | **+** | **-** | **+** | **-** | **?** | **+** |  |  |
| **Kanwal, 2017** | **?** | **-** | **+** | **-** | **+** | **+** | **-** | **-** |  |  |
| **Ioannou, 2017** | **+** | **?** | **+** | **+** | **+** | **+** | **?** | **+** |  |  |
| **Tachi, 2017** | **+** | **+** | **+** | **-** | **+** | **+** | **+** | **+** |  |  |
| **Nagaoki, 2017** | **+** | **-** | **+** | **+** | **+** | **+** | **+** | **+** |  |  |
| **Backus, 2017** | **+** | **-** | **+** | **-** | **+** | **+** | **-** | **-** |  |  |
| **Kozbial, 2017** | **+** | **+** | **+** | **-** | **+** | **+** | **+** | **+** |  |  |
| **Pascasio, 2017** | **+** | **-** | **+** | **+** | **+** | **+** | **+** | **+** |  |  |
| **Belli, 2017** | **+** | **+** | **+** | **-** | **-** | **+** | **?** | **?** |  |  |
| **nunez, 2017** | **+** | **+** | **+** | **-** | **-** | **+** | **?** | **+** |  |  |
| **Li, 2018** | **+** | **+** | **+** | **+** | **+** | **+** | **-** | **-** |  |  |
| **Romano, 2017** | **+** | **+** | **+** | **+** | **+** | **+** | **+** | **+** |  |  |
| **Kondili, 2017** | **+** | **?** | **+** | **+** | **+** | **+** | **+** | **?** |  |  |
| **Sun Hong Yoo, 2017** | **-** | **-** | **+** | **-** | **+** | **-** | **+** | **+** |  |  |
| **Mangia, 2017** | **+** | **+** | **+** | **+** | **+** | **+** | **+** | **+** |  |  |
| **Sangiovanni, 2017** | **+** | **+** | **+** | **-** | **+** | **+** | **+** | **?** |  |  |
| **Miyase, 2017** | **-** | **-** | **+** | **+** | **+** | **+** | **+** | **+** |  |  |
| **kuftinec, 2017** | **+** | **+** | **+** | **-** | **+** | **+** | **+** | **+** |  |  |
| **Lleo, 2018** | **+** | **+** | **+** | **-** | **+** | **+** | **+** | **+** |  |  |
| **DAA occurrence & recurrence studies** |  |  |  |  |  |  |  |  |  |  |
| **Conti, 2016** | **+** | **+** | **+** | **-** | **+** | **-** | **+** | **+** | **+** | **+** |
| **Lei-Zeng, 2016** | **+** | **?** | **+** | **-** | **+** | **+** | **+** | **+** | **?** | **+** |
| **Cheung, 2016** | **+** | **-** | **+** | **+** | **-** | **+** | **-** | **?** | **+** | **-** |
| **Issachar, 2017** | **-** | **?** | **+** | **-** | **?** | **+** | **+** | **+** | **?** | **?** |
| **Rinaldi, 2016** | **+** | **+** | **+** | **-** | **+** | **-** | **+** | **+** | **+** | **+** |
| **Menzaghi, 2017** | **-** | **-** | **+** | **+** | **+** | **+** | **?** | **?** | **?** | **?** |
| **Douhara, 2017** | **?** | **+** | **+** | **-** | **+** | **-** | **+** | **+** | **-** | **+** |
| **Cajella, 2017** | **+** | **-** | **+** | **-** | **+** | **-** | **?** | **+** | **+** | **-** |
| **Murcia, 2017** | **?** | **?** | **+** | **-** | **+** | **+** | **?** | **?** | **+** | **+** |
| **Fangazio, 2017** | **?** | **+** | **+** | **-** | **+** | **-** | **+** | **+** | **-** | **-** |
| **Donato, 2017** | **+** | **?** | **+** | **+** | **+** | **+** | **+** | **+** | **?** | **+** |
| **Bielen, 2017** | **+** | **+** | **+** | **+** | **+** | **-** | **+** | **+** | **+** | **+** |
| **Degasperi, 2017** | **+** | **+** | **+** | **-** | **-** | **+** | **+** | **+** | **-** | **-** |
| **Ogawa, 2017** | **+** | **?** | **+** | **-** | **+** | **+** | **+** | **?** |  |  |
| **Ponziani, 2017** | **+** | **-** | **+** | **-** | **+** | **+** | **+** | **+** | **-** | **-** |
| **Sterling, 2018** | **+** | **+** | **+** | **-** | **+** | **-** | **+** | **+** | **-** | **-** |

**Table S1: Risk of bias assessment of the included DAA studies (using the Cochrane tool for risk of bias).**

+ : low risk of bias

? : unclear risk of bias

- : high risk of bias

*: if the included subjects had undergone imaging which could assess for HCC within six months of starting treatment, the study was considered low risk for bias

#: studies with follow-up periods of one year or greater were considered low risk for bias

^: studies with at least a six month interval between initial HCC curative treatment and initiation of HCV treatment were considered low risk for bias

HCC= hepatocellular carcinoma

HCV= hepatitis C virus

DAA= direct-acting antiviral

IFN= interferon
