# Supplementary material for: No evidence for higher rates of hepatocellular carcinoma after direct-acting antiviral treatment: a meta-analysis
Source: Hepatoma Res. Author manuscript; Available in PMC 2019 Aug 26. (PMC6709867; doi:10.20517/2394-5079.2019.19)
Supplement: Supplemental table2 [file NIHMS1046854-supplement-Supplemental_table2.docx]

| **Study** | **Ascertainment of cirrhosis** | **Time since last imaging** | **Ascertainment of SVR** | **Control group/comparator** | **Adjusted for potential confounders** | **Follow-up duration** | **Assessment during follow-up** | **Assessment of HCC event** | **Time interval between HCC and initiation of HCV therapy** | **Data on initial HCC management** |
| --- | --- | --- | --- | --- | --- | --- | --- | --- | --- | --- |
| **IFN Recurrence Studies** |  |  |  |  |  |  |  |  |  |  |
| **Hagihara, 2011** |  |  | **+** | **+** | **+** | **+** | **+** | **+** | **+** | **+** |
| **Kanogawa, 2015** |  |  | **+** | **+** | **+** | **+** | **+** | **+** | **?** | **+** |
| **Kunimoto, 2016** |  |  | **+** | **+** | **+** | **+** | **+** | **+** | **?** | **+** |
| **Jeong, 2007** |  |  | **+** | **+** | **+** | **+** | **+** | **+** | **+** | **+** |
| **Saito, 2014** |  |  | **+** | **+** | **+** | **+** | **+** | **+** | **+** | **+** |
| **Sanefuji, 2009** |  |  | **+** | **+** | **+** | **+** | **+** | **+** | **+** | **+** |
| **Minami, 2016** |  |  | **+** | **+** | **+** | **+** | **?** | **?** | **+** | **+** |
| **Petta, 2017** |  |  | **+** | **+** | **+** | **+** | **+** | **+** | **-** | **+** |
| **Bruno 2017** |  |  | **+** | **+** | **+** | **+** | **+** | **-** | **-** | **+** |
| **Hsu, 2013** |  |  | **-** | **+** | **+** | **+** | **+** | **+** | **-** | **+** |
| **Urabe, 2017** |  |  | **-** | **+** | **+** | **+** | **+** | **?** | **+** | **-** |
| **Kudo, 2007** |  |  | **-** | **+** | **+** | **+** | **+** | **+** | **-** | **+** |
| **Mazzaferro, 2006** |  |  | **+** | **+** | **+** | **+** | **+** | **+** | **-** | **+** |
| **Tanimoto, 2012** |  |  | **+** | **+** | **+** | **+** | **+** | **+** | **-** | **+** |
| **Shirtatori, 2003** |  |  | **+** | **+** | **+** | **+** | **+** | **+** | **-** | **+** |
| **Kubo, 2001** |  |  | **+** | **+** | **+** | **+** | **+** | **+** | **-** | **+** |
| **IFN Occurrence Studies** |  |  |  |  |  |  |  |  |  |  |
| **Miyase, 2017** | **-** | **-** | **+** | **+** | **+** | **?** | **+** | **+** |  |  |
| **Yamashita, 2014** | **+** | **+** | **+** | **-** | **+** | **+** | **+** | **+** |  |  |
| **Ogawa, 2013** | **+** | **-** | **+** | **-** | **+** | **+** | **+** | **+** |  |  |
| **van der Meer, 2017** | **+** | **-** | **+** | **-** | **+** | **+** | **?** | **+** |  |  |
| **Bruno, 2009** | **+** | **+** | **+** | **+** | **+** | **+** | **+** | **+** |  |  |
| **Mallet, 2008** | **+** | **+** | **+** | **+** | **+** | **+** | **+** | **+** |  |  |
| **Cardoso, 2010** | **+** | **?** | **+** | **+** | **+** | **+** | **+** | **+** |  |  |
| **Yu, 2006** | **+** | **+** | **+** | **+** | **+** | **+** | **+** | **+** |  |  |
| **Hung, 2006** | **+** | **+** | **+** | **+** | **+** | **+** | **+** | **+** |  |  |
| **Morgan, 2010** | **+** | **+** | **+** | **+** | **+** | **+** | **+** | **+** |  |  |
| **Aleman, 2013** | **+** | **?** | **+** | **+** | **+** | **+** | **+** | **+** |  |  |
| **Cheinquer, 2010** | **+** | **+** | **+** | **+** | **+** | **+** | **+** | **+** |  |  |
| **Moon, 2015** | **+** | **+** | **+** | **+** | **+** | **+** | **+** | **+** |  |  |
| **Fernandez-Rodriguez, 2010** | **+** | **+** | **+** | **+** | **+** | **+** | **+** | **+** |  |  |
| **Janjua, 2016** | **+** | **?** | **+** | **+** | **+** | **+** | **?** | **+** |  |  |
| **Rutter, 2016** | **+** | **+** | **+** | **+** | **+** | **+** | **+** | **+** |  |  |
| **Velosa, 2011** | **+** | **+** | **+** | **+** | **+** | **+** | **+** | **+** |  |  |
| **Nahon, 2017** | **+** | **+** | **+** | **+** | **+** | **+** | **+** | **+** |  |  |
| **Di Marco, 2016** | **+** | **+** | **+** | **+** | **+** | **+** | **+** | **+** |  |  |
| **Trapero-Marugan, 2011** | **+** | **-** | **+** | **-** | **+** | **+** | **+** | **+** |  |  |
| **El Braks, 2007** | **+** | **+** | **+** | **-** | **+** | **+** | **+** | **+** |  |  |
| **van der Meer, 2012** | **+** | **-** | **+** | **-** | **+** | **+** | **+** | **+** |  |  |
| **El-Serag, 2016** | **+** | **-** | **+** | **-** | **+** | **+** | **+** | **?** |  |  |
| **Kobayashi, 2017** | **+** | **-** | **+** | **+** | **+** | **+** | **+** | **+** |  |  |
| **Toyoda, 2016** | **+** | **-** | **+** | **+** | **+** | **+** | **?** | **?** |  |  |
| **Innes, 2017** | **-** | **?** | **+** | **+** | **+** | **+** | **-** | **-** |  |  |
| **Ioannou, 2017** | **+** | **?** | **+** | **+** | **+** | **+** | **?** | **+** |  |  |
| **Nagaoki, 2017** | **+** | **-** | **+** | **+** | **+** | **+** | **+** | **+** |  |  |
| **Li, 2018** | **+** | **+** | **+** | **+** | **+** | **+** | **-** | **-** |  |  |
| **Miyase, 2017** | **-** | **-** | **+** | **+** | **+** | **+** | **+** | **+** |  |  |
| **Hsu, 2013** | **-** | **-** | **-** | **+** | **+** | **+** | **+** | **+** |  |  |
| **Cardoso, 2016** | **+** | **?** | **+** | **?** | **+** | **+** | **?** | **?** |  |  |

**Table S2: Risk of bias assessment of the included IFN studies (using the Cochrane tool for risk of bias).**

+ : low risk of bias

? : unclear risk of bias

- : high risk of bias

*: if the included subjects had undergone imaging which could assess for HCC within six months of starting treatment, the study was considered low risk for bias

#: studies with follow-up periods of one year or greater were considered low risk for bias

^: studies with at least a six month interval between initial HCC curative treatment and initiation of HCV treatment were considered low risk for bias

HCC= hepatocellular carcinoma

HCV= hepatitis C virus

DAA= direct-acting antiviral

IFN= interferon
