# Supplementary material for: No evidence for higher rates of hepatocellular carcinoma after direct-acting antiviral treatment: a meta-analysis
Source: Hepatoma Res. Author manuscript; Available in PMC 2019 Aug 26. (PMC6709867; doi:10.20517/2394-5079.2019.19)
Supplement: Supplemental table3 [file NIHMS1046854-supplement-Supplemental_table3.docx]

| **Study, year** | **Country** | **Setting** | **Cohort Design** | **Data source** | **Patient,n** | **Age (years)** | **Male (%)** | **GT 1 (%)** | **GT 3 (%)** | **Cirrhosis (%)** | **CPA (%)** | **Follow-up (years)** | **SVR** | **De Novo Num** | **De Novo Denom** | **Occurrence IR per 100 person years** | **Recurrence Num** | **Recurrence Denom** | **IR recurren-ce per 100 py** |
| --- | --- | --- | --- | --- | --- | --- | --- | --- | --- | --- | --- | --- | --- | --- | --- | --- | --- | --- | --- |
| **Recurrence** |  |  |  |  |  |  |  |  |  |  |  |  |  |  |  |  |  |  |  |
| **Yang, 2016** | US | Multi-center | Retrospective | Report | 18 |  |  |  |  |  |  |  | 50% |  |  |  | 5 | 18 |  |
| **Pol, CO22,2016** | France | Multi-center | Prospective | Full article | 189 | 62 | 78.0 | 65.0 |  | 77.5 |  | 1.68 | 92% |  |  |  | 24 | 189 | 7.54361 |
| **Pol, CO12, 2016** | France | Multi-center | Prospective | Full article | 13 | 61 | 85.0 | 85.0 | 7.9 | 100.0 | 100.0 | 1.78 | 100% |  |  |  | 1 | 13 | 4.33369 |
| **Pol, CO23, 2016** | France | Multi-center | Prospective | Full article | 314 | 61 | 82.0 | 67.5 |  | 0.0 |  | 0.58 | 97% |  |  |  | 7 | 314 | 3.82187 |
| **Zavaglia, 2017** | Italy | Multi-center | Prospective | Brief report | 31 | 65 | 64.5 | 87.1 |  | 100.0 | 81.0 | 0.67 | 84% |  |  |  | 1 | 31 | 4.83871 |
| **Torres, 2016** | US | Single-center | Prospective | Brief report | 8 | 64 | 87.5 | 75.0 |  | 88.5 | 37.5 | 1.60 | 75% |  |  |  |  |  |  |
| **Adhoute, 2018** | France | Single-center | Retrospective  (case-control) | Full article | 22 | 63 | 73.0 | 82.0 | 13.0 | 100.0 | 82.0 | 5.67 | 86% |  |  |  |  |  |  |
| **Petta, 2017** | Italy | Multi-center | Retrospective  (post-hoc analysis) | Full article | 58 | 66.3 | 69.0 |  |  | 100.0 | 91.0 | 1.50 |  |  |  |  | 16 | 58 | 18.3908 |
| **Minami, 2016** | Japan | Single-center | Retrospective | Brief report | 27 | 71 | 67.0 | 78.0 | 0.0 | 100.0 | 100.0 | 1.30 | 85% |  |  |  | 8 | 26 | 23.6686 |
| **Reig, 2017** | Spain | Multi-center | Prospective | Full article | 77 | 66 | 69.0 | 91.0 | 3.4 | 94.8 | 91.0 | 0.68 | 95% |  |  |  | 21 | 77 | 39.9113 |
| **Huang, 2017** | US | Single-center | Retrospective | conference | 178 | 62 | 74.0 | 72.5 | 22.7 | 100.0 | 52.8 |  |  |  |  |  |  |  |  |
| **Ikeda, 2017** | Japan | Single-center | Prospective | Full article | 177 | 71 | 59.9 | 93.2 | 0.0 |  | 96.8 | 1.73 | 90% |  |  |  | 61 | 177 | 19.9787 |
| **Cabibbo, RESIST, 2017** | Italy | Multi-center | Prospective | Full article | 143 | 70.4 | 60.1 | 86.0 | 4.9 | 100.0 | 86.0 | 0.73 | 97% |  |  |  | 29 | 143 | 27.972 |
| **Kolly, 2017** | Europe | Multi-center | Retrospective | Brief report | 47 | 60 | 76.6 | 53.2 | 31.9 | 85.1 | 80.0 | 1.79 |  |  |  |  | 20 | 47 | 23.7506 |
| **Virlogeuz, 2017** | France | Single-center | Prospective | Full article | 23 | 58 | 87.0 | 62.0 | 24.0 | 100.0 | 87.0 | 2.34 | 96% |  |  |  | 11 | 23 | 20.424 |
| **Sangiovanni, 2017** | Italy | Multi-center | Prospective | Abstract | 89 | 69 | 65.0 |  |  | 100.0 |  | 0.87 | 96% |  |  |  | 24 | 80 | 34.6667 |
| **Minami, 2017** | Japan | Single-center | Retrospective | Abstract | 163 | 74 | 58.9 |  |  |  |  | 1.21 | 92% |  |  |  | 78 | 163 | 39.6023 |
| **Urabe, 2018** | Japan | Multi-center | Prospective | Abstract | 63 |  |  |  |  |  |  | 0.91 |  |  |  |  | 24 | 63 | 41.9397 |
| **Lleo, 2018** | Italy | Multi-center | Prospective | Full article | 161 | 65.8 | 68.9 | 70.8 | 12.4 | 100.0 | 86.3 | 0.95 | 95% |  |  |  | 38 | 161 | 24.8 |
| **Occurrence** |  |  |  |  |  |  |  |  |  |  |  |  |  |  |  |  |  |  |  |
| **Romano, 2018** | Italy | Multi-center | Prospective | Full article | 3917 | 58.1 | 62.2 | 61.8 | 16.8 | 70.0 | 91.0 | 1.47 | 94% | 55 | 3917 | 0.95519 |  |  |  |
| **Kobayashi, 2017** | Japan | Single-center | Retrospective | Full article | 77 | 63 | 44.2 | 100.0 |  | 0.0 |  | 4.00 | 100% | 2 | 77 | 0.64935 |  |  |  |
| **Cardoso, 2016** | Portugal | Single-center | Retrospective | Brief report | 54 | 59 | 70.4 | 78.0 | 18.5 | 100.0 | 65.0 | 1.69 | 100% | 4 | 54 | 4.37712 |  |  |  |
| **Toyoda, 2016** | Japan | Single-center | Retrospective | Brief report | 413 |  |  |  |  | 37.8 |  | 1.00 | 100% | 3.5 | 413 | 0.84746 |  |  |  |
| **Mangia, 2016** | Italy | Single-center | Prospective | Abstract | 1288 | 65.7 | 56.0 | 55.0 | 10.0 | 49.0 | 95.7 | 1.00 | 96% | 5 | 1206 | 0.41459 |  |  |  |
| **Innes, 2018** | Scotland | Multi-center | Prospective | Full article | 272 | 52.1 | 71.3 |  | 27.2 | 100.0 | 69.6 | 1.70 | 100% | 12 | 272 | 2.59516 |  |  |  |
| **Ji, 2017** | China | Single-center | Prospective | Abstract | 165 | 51 | 51.0 | 82.2 |  | 48.0 |  | 1.17 | 100% |  |  |  |  |  |  |
| **Korenaga, 2018** | Japan | Multi-center | propsective | Abstract | 509 |  |  | 100.0 |  | 23.0 |  | 4.00 | 100% | 9 | 509 | 0.44204 |  |  |  |
| **Calvaruso, 2018** | Sicily | Multi-center | Prospective | Full article | 2249 | 65.4 | 56.9 | 79.9 | 6.8 | 77.8 | 68.6 | 1.17 | 95% | 78 | 2249 | 2.97275 |  |  |  |
| **Ravi, 2017** | US | Single-center | Retrospective | Brief report | 66 | 60 | 62.0 | 71.0 |  | 100.0 |  | 0.50 | 92% | 6 | 66 | 18.1818 |  |  |  |
| **Muir, 2018** | US | Multi-center | Prospective | Conference | 1564 | 59 | 68.0 |  |  | 100.0 | 85.0 | 1.02 | 100% | 55 | 1564 | 3.45027 |  |  |  |
| **Sogni, CO13, 2016** | France | Multi-center | Prospective | Full article | 189 | 53.2 | 74.6 | 58.0 | 17.0 | 100.0 | 91.0 | 0.23 | 93% | 2 | 189 | 4.58554 |  |  |  |
| **Kanwal, 2017** | US | Multi-center | Retrospective | Full article | 22500 | 61.6 | 96.7 | 86.8 | 4.2 | 39.0 |  | 1.02 | 87% | 271 | 22500 | 1.18016 |  |  |  |
| **Ioannou, 2017** | US | Multi-center | Retrospective | Full article | 21948 | 61 | 96.8 | 84.6 | 4.9 | 23.8 |  | 1.53 | 91% | 455 | 23090 | 1.32 |  |  |  |
| **Nagaoki, 2017** | Japan | Single-center | Retrospective | Full article | 154 | 73 | 37.7 | 100.0 |  | 53.2 |  | 1.92 | 100% | 7 | 154 | 2.37154 |  |  |  |
| **Backus, 2017** | US | Multi-center | Retrospective | Full article | 14024 | 63.1 | 97.2 | 85.0 | 6.6 | 100.0 |  | 1.62 | 93% | 537 | 14024 | 2.36529 |  |  |  |
| **Kozbial, 2017** | Austria | Multi-center | Retrospective | Full article | 551 | 57.9 | 61.2 | 86.4 | 7.3 | 71.5 | 86.2 | 1.26 | 96% | 13 | 551 | 1.87021 |  |  |  |
| **Pascasio, 2017** | Spain | Multi-center | Retrospective | Full article | 29 | 55 | 79.0 | 73.0 | 17.0 | 100.0 | 31.0 | 1.88 | 84% | 1 | 29 | 1.83594 |  |  |  |
| **Belli, 2017** | France | Single-center | Prospective | abstract | 34 |  |  |  |  |  |  | 2.13 |  | 0 | 34 | 0 |  |  |  |
| **nunez, 2017** | Spain | Single-center | Retrospective | Abstract | 339 | 58 | 63.0 | 81.0 | 11.0 | 86.1 | 78.0 | 1.10 |  | 8 | 339 | 2.15287 |  |  |  |
| **Li, 2018** | US | Multi-center | Retrospective | Full article | 5834 | 62 | 96.6 | 84.5 | 3.3 | 19.9 |  | 1.08 | 96% | 50 | 5834 | 0.792 |  |  |  |
| **Romano, 2017** | Italy | Single-center | Prospective | Abstract | 93 |  |  |  |  | 100.0 | 77.4 | 1.27 |  |  |  |  |  |  |  |
| **Kondili, 2017** | Italy | Multi-center | Prospective | Abstract | 1457 | 60 | 95.0 |  |  | 55.7 |  | 1.00 | 95% | 0 | 811 | 0 |  |  |  |
| **Sun Hong Yoo, 2017** | Korea | Multi-center | Retrospective | Abstract | 574 |  |  |  |  |  |  | 0.87 | 96% | 5 | 574 | 1.00509 |  |  |  |
| **Mangia, 2017** | Italy | Single-center | Prospective | Abstract | 1170 | 61.9 | 60.0 |  |  | 48.0 | 89.0 | 1.78 |  | 14 | 1170 | 0.67098 |  |  |  |
| **Sangiovanni, 2017** | Italy | Multi-center | Prospective | Abstract | 505 | 63 | 62.0 |  |  | 100.0 |  | 1.00 | 95% | 20 | 505 | 3.9604 |  |  |  |
| **Miyase, 2017** | Japan | Single-center | Prospective | Abstract | 516 |  |  |  |  |  |  |  |  |  |  | 0.83 |  |  |  |
| **kuftinec, 2017** | US | Single-center | Prospective | Abstract | 153 |  |  |  |  |  |  | 1.00 | 86% | 7 | 153 | 4.57516 |  |  |  |
| **Lleo, 2018** | Italy | Multi-center | Prospective | Full article | 1766 | 61.7 | 61.9 | 67.6 | 12.3 | 100.0 | 88.6 | 1.18 | 95% | 50 | 1766 | 2.4 |  |  |  |
| **Cardoso, 2016** | Portugal | Single-center | Retrospective | Brief report | 54 | 59 | 70.0 | 78.0 | 18.0 | 100.0 | 64.8 | 1.00 | 100% | 4 | 54 |  |  |  |  |
| **Occurrence & recurrence** |  |  |  |  |  |  |  |  |  |  |  |  |  |  |  |  |  |  |  |
| **Conti, 2016** | Italy | Multi-center | Retrospective | Full article | 344 | 63 | 60.2 | 68.9 | 11.0 | 100.0 | 88.7 | 0.46 | 91% | 9 | 285 | 6.84268 | 17 | 59 | 62.4346 |
| **Lei-Zeng, 2016** | China | Single-center | Prospective | Brief report | 31 | 56 | 19.0 | 100.0 | 0.0 | 100.0 | 67.0 | 1.25 | 97% | 0 | 21 |  | 0 | 10 |  |
| **Cheung, 2016** | England | Multi-center | Prospective | Full article | 406 | 54 | 0.0 | 48.8 | 42.1 | 100.0 | 17.2 | 1.25 | 78% | 25 | 377 | 5.30504 | 2 | 29 | 5.51724 |
| **Issachar, 2017** | Israel | Single-center | Retrospective | Conference | 273 | 58 | 50.9 | 88.3 | 5.1 | 53.1 | 100.0 | 1.50 | 95% | 6 | 260 | 1.53846 | 3 | 13 | 15.3846 |
| **Rinaldi, 2016** | Italy | Multi-center | Prospective | Full article | 265 | 68 | 53.0 | 76.0 |  | 100.0 | 86.8 | 0.23 | 97% | 8 | 265 | 13.0818 | 1 | 15 | 28.8889 |
| **Menzaghi, 2017** | Italy | Multi-center | Prospective | Conference | 1125 | 56.2 | 69.2 | 61.3 | 18.5 |  |  | 1.39 | 94% | 21 | 1125 | 1.34132 | 6 | 29 | 14.8668 |
| **Douhara, 2017** | Japan | Single-center | Retrospective | Full article | 33 | 68 | 54.5 | 81.8 | 0.0 | 24.2 |  | 0.87 | 100% | 0 | 32 |  | 1 | 1 | 115.385 |
| **Cajella, 2017** | Spain | Multi-center | Retrospective | Full article | 1567 | 59.5 | 53.7 | 100.0 | 0.0 | 46.7 | 98.6 | 0.48 | 97% | 30 | 3233 | 1.92302 | 21 | 70 | 62.1712 |
| **Murcia, 2017** | Spain | Multi-center | Prospective | Brief report | 179 | 59 | 56.2 | 79.0 |  | 100.0 | 70.0 | 1.75 | 98% | 11 | 179 | 3.51157 | 2 | 14 | 8.16327 |
| **Fangazio, 2017** | Italy | Single-center | Prospective | Brief report | 286 | 64 | 58.0 |  |  | 66.0 |  | 0.46 | 94% | 9 | 273 | 7.14286 | 2 | 13 | 33.3333 |
| **Donato, 2017** | Italy | Single-center | Prospective | Brief report | 35 | 57 | 83.0 |  |  | 100.0 |  | 1.67 | 94% | 0 | 16 |  | 3 | 35 | 5.14286 |
| **Bielen, 2017** | Belgium | Multi-center | Retrospective | Full article | 490 | 59 | 62.7 | 69.9 | 14.1 | 67.0 | 100.0 | 0.50 | 94% | 4 | 355 | 2.25352 | 6 | 40 | 30 |
| **Degasperi, 2019** | Italy | Single-center | Prospective | Full article | 565 | 64 | 60.0 | 49.0 | 11.0 | 100.0 | 87.0 | 2.08 for occurrence; 1.92 for recurrence | 97% | 20 | 517 | 1.85687 | 9 | 48 | 9.78261 |
| **Ogawa, 2017** | Japan | Multi-center | Prospective | Abstract | 1675 |  |  |  |  |  |  | 1.42 |  | 18 | 1523 | 0.83427 | 25 | 152 | 11.6099 |
| **Ponziani, 2017** | Italy | Single-center | Prospective | Abstract | 185 |  |  |  |  | 83.2 | 94.6 | 1.32 | 95% | 2 | 130 | 1.16845 | 17 | 24 | 53.7975 |
| **Sterling, 2018** | US | Single-center | Prospective | Abstract | 342 | 58 | 60.0 | 89.0 |  |  |  | 0.50 | 91% | 6 | 321 | 3.73832 | 4 | 21 | 38.0952 |
| **Tachi, 2017** | Japan | Single-center | Prospective | Full article | 263 | 70.7 | 46.4 | 75.6 |  | 18.6 | 100.0 | 1.51 | 100% | 7 | 233 | 1.9918 | 11 | 30 |  |

**Table S3. Names of DAA studies included and characteristics of patients in these studies.**

| **Mean** |
| --- |
| **Exact number for all subjects** |
| **Median** |

**Notes on individual studies**

For Innes, median and mean f/u are both 1.7 yrs. Median time to event is 1 year, median is 0.9 years

Korenaga included patients co-infected with HBV (1.4%) and HIV (2.8%). ~5% patients >12 months US prior to DAA but mean <6 months

Menzaghi included 34% co-infected patients with HIV

In CO13, 100% of patients were co-infected with HIV

Kanwal, Ioannou, Backus, Li were VA studies

Murcia, time to HCC is for recurrence arm

Ioannou includes 4% HIV co-infection, 1% HBV co-infection. Excluded anyone w/ <180 days of follow-up

Bielen included 5.3% HIV co-infected and 1.4% HBV co-infected

In Sterling, 5% were HIV co-infected

Denom = denominator

GT = genotype

HBV = hepatitis B

IR = incidence rate

Num = numerator

VA = Veterans Affairs
