# Supplementary material for: No evidence for higher rates of hepatocellular carcinoma after direct-acting antiviral treatment: a meta-analysis
Source: Hepatoma Res. Author manuscript; Available in PMC 2019 Aug 26. (PMC6709867; doi:10.20517/2394-5079.2019.19)
Supplement: Supplemental table4 [file NIHMS1046854-supplement-Supplemental_table4.docx]

| **Study, year** | **Country** | **Setting** | **Cohort Design** | **Data source** | **Patient,n** | **Age (years)** | **Male (%)** | **GT 1 (%)** | **GT 3 (%)** | **Cirrhosis (%)** | **CPA (%)** | **Follow-up (years)** | **SVR** | **De Novo Num** | **De Novo Denom** | **Occurrence IR per 100 person years** | **Recurrence Num** | **Recurrence Demon** | **IR recurrence per 100 py** |
| --- | --- | --- | --- | --- | --- | --- | --- | --- | --- | --- | --- | --- | --- | --- | --- | --- | --- | --- | --- |
| **Recurrence** |  |  |  |  |  |  |  |  |  |  |  |  |  |  |  |  |  |  |  |
| **Hagihara, 2011** | Japan | Single-center | Retrospective | Full article | 37 | 63 | 78.0 | 62.2 |  |  |  | 4.60 | 51.4% |  |  |  | 18 | 37 | 10.58 |
| **Kanogawa, 2015** | Japan | Multi-center | Retrospective | Full article | 41 | 65 | 76.0 | 47.6 |  | 100.0 | 100.0 | 7.01 | 53.7% |  |  |  | 22 | 41 | 7.66 |
| **Kunimoto, 2016** | Japan | Single-center | Retrospective | Full article | 120 | 65 | 88.0 | 72.0 |  | 35.0 |  | 5.08 | 100.0% |  |  |  | 16 | 40 | 7.87 |
| **Jeong, 2007** | Japan | Single-center | Retrospective | Full article | 42 | 62 | 85.7 | 66.7 | 0.0 | 92.9 | 83.3 | 2.67 | 69.0% |  |  |  | 20 | 42 | 17.86 |
| **Saito, 2014** | Japan | Multi-center | Retrospective | Full article | 51 | 72 | 92.9 | 44.4 |  | 14.3 |  | 3.45 | 27.5% |  |  |  | 39 | 51 | 22.17 |
| **Sanefuji, 2009** | Japan | Single-center | Prospective | Full article | 26 | 68 | 65.4 |  |  |  | 100.0 | 3.75 |  |  |  |  | 3 | 6 | 13.33 |
| **Minami, 2016** | Japan | Single-center | Prospective | Brief report | 38 | 66 | 71.0 | 76.0 |  |  | 100.0 | 7.60 | 34.2% |  |  |  | 26 | 38 | 9.00 |
| **Petta, 2017** | Italy | Multi-center | Retrospective | Full article | 57 | 62 | 72.0 |  |  | 100.0 | 90.0 | 2.83 | 100.0% |  |  |  | 22 | 57 | 13.62 |
| **Bruno 2017** | Italy | Multi-center | Retrospective | Full article | 175 |  | 78.9 | 79.3 |  | 100.0 | 88.9 | 3.00 | 10.9% |  |  |  |  |  |  |
| **Hsu, 2013** | Taiwan | Multi-center | Retrospective | Full article | 213 | 62.1 | 66.2 |  |  | 38.5 |  | 2.01 |  |  |  |  | 62 | 213 | 14.48 |
| **Kudo, 2007** | Japan | Single-center | Prospective matched case-control study | Full article | 43 | 65 | 76.7 | 100.0 |  |  | 100.0 | 5.10 | 4.7% |  |  |  | 24 | 43 | 10.94 |
| **Mazzaferro, 2006** | Italy | Multi-center | Randomized controlled trial | Full article | 42 | 65 | 83.3 | 75.3 | 3.3 | 100.0 | 97.6 | 3.75 | 4.8% |  |  |  | 48 | 76 | 16.84 |
| **Tanimoto, 2012** | Japan | Single-center | Prospective, propensity score analysis/matching | Full article | 38 | 65 .5 | 60.5 | 74.4 | 0.0 |  | 1.0 | 3.80 | 42.1% |  |  |  | 21 | 38 | 14.54 |
| **Shirtatori, 2003** | Japan | Single-center | Randomized controlled trial | Full article | 49 | 61 | 71.4 | 61.2 | 0.0 | 100.0 | 100.0 | 7.10 | 28.6% |  |  |  | 37 | 49 | 10.64 |
| **Kubo, 2001** | Japan | Single-center | Randomized controlled trial | Brief report | 15 | 61.9 | 1.0 | 80.0 | 0.0 | 47.0 | 73.3 | 2.98 | 13.3% |  |  |  | 5 | 15 | 11.20 |
| **Urabe, 2017** | Japan | Multi-center | Prospective | Abstract | 56 |  |  |  |  |  |  | 2.83 |  |  |  |  | 23 | 56 | 14.54 |
| **Occurrence** |  |  |  |  |  |  |  |  |  |  |  |  |  |  |  |  |  |  |  |
| **Miyase, 2017** | Japan | Single-center | Retrospective | Abstract | 524 |  |  |  |  |  |  |  | 100.0% |  |  | 0.84 |  |  |  |
| **Yamashita, 2014** | Japan | Single-center | Retrospective | Full article | 562 | 57 | 55.3 | 41.8 |  | 9.0 |  | 4.80 | 100.0% | 31 | 562 | 1.15 |  |  |  |
| **Ogawa, 2013** | Japan | Multi-center | Prospective | Full article | 1013 | 58 | 49.2 | 70.1 | 0.0 | 14.8 |  | 3.60 | 55.0% | 47 | 1013 | 1.29 |  |  |  |
| **van der Meer, 2017** | The Netherlands | Multi-center | Retrospective pooled cohort studies | Full article | 1000 | 52.7 | 67.8 | 50.0 | 19.7 | 85.0 | 100.0 | 5.70 | 100.0% | 51 | 1000 | 0.89 |  |  |  |
| **Bruno, 2009** | Italy | Multi-center | Prospective | Full article | 194 | 59 | 51.0 |  |  | 100.0 | 100.0 | 8.01 | 14.0% | 56 | 194 | 3.60 |  |  |  |
| **Mallet, 2008** | France | Single-center | Prospective | Brief report | 96 | 45 | 60.0 | 53.1 |  | 100.0 | 100.0 | 9.83 | 36.5% | 17 | 96 | 1.80 |  |  |  |
| **Cardoso, 2010** | France | Single-center | Retrospective | Full article | 307 | 55 | 67.0 | 60.0 | 16.0 | 58.0 |  | 3.50 | 33.6% | 46 | 307 | 4.28 |  |  |  |
| **Yu, 2006** | Taiwan | Multi-center | Retrospective-prospective | Full article | 1057 | 46.9 | 60.5 | 45.7 |  | 15.6 |  | 5.18 | 67.6% | 51 | 1057 | 0.93 |  |  |  |
| **Hung, 2006** | Taiwan | Single-center | Prospective | Full article | 132 | 56 | 52.0 | 50.4 |  | 100.0 | 100.0 | 3.08 | 55.3% | 16 | 132 | 3.93 |  |  |  |
| **Morgan, 2010** | USA | Multi-center | Prospective | Full article | 526 | 49 | 72.0 | 87.0 | 5.1 | 35.0 |  | 6.90 | 26.6% | 35 | 526 | 0.96 |  |  |  |
| **Aleman, 2013** | Sweden | Multi-center | Prospective | Full article | 303 | 51 | 69.0 | 50.0 |  | 100.0 | 100.0 | 5.40 | 36.3% | 32 | 303 | 1.96 |  |  |  |
| **Cheinquer, 2010** | Brazil | Single-center | Prospective | Full article | 85 | 52 | 66.0 | 29.6 |  | 100.0 | 100.0 | 2.50 | 45.0% | 9 | 85 | 4.24 |  |  |  |
| **Moon, 2015** | Korea | Single-center | Retrospective | Full article | 463 | 50.9 | 47.7 | 57.7 | 1.7 | 100.0 | 100.0 | 3.01 | 64.8% | 12 | 463 | 0.86 |  |  |  |
| **Fernandez-Rodriguez, 2010** | Spain | Multi-center | Retrospective | Full article | 568 | 51 | 69.4 | 70.1 |  | 100.0 | 100.0 | 2.92 | 30.6% | 31 | 568 | 1.87 |  |  |  |
| **Janjua, 2016** | Canada | Registry | Retrospective | Full article | 8147 | 50 | 67.6 | 46.7 | 25.1 | 5.0 |  | 5.60 | 57.2% | 174 | 8147 | 0.38 |  |  |  |
| **Rutter, 2016** | Austria | Single-center | Prospective | Full article | 714 | 51.4 | 61.3 | 64.1 | 11.5 | 100.0 |  | 7.20 | 77.2% | 29 | 714 | 0.56 |  |  |  |
| **Velosa, 2011** | Portugal | Single-center | Prospective | Full article | 130 | 51.7 | 70.8 | 61.0 |  | 100.0 | 100.0 | 6.40 | 30.0% | 21 | 130 | 2.52 |  |  |  |
| **Nahon, 2017** | France | Multi-center | Prospective | Full article | 1323 | 55 | 63.0 | 67.9 | 15.6 | 100.0 | 100.0 | 4.85 | 50.6% | 182 | 1323 | 2.84 |  |  |  |
| **Di Marco, 2016** | Italy | Single-center | Prospective | Full article | 444 | 58 | 62.2 | 83.4 | 3.8 | 100.0 |  | 7.60 | 24.3% | 99 | 444 | 2.93 |  |  |  |
| **Trapero-Marugan, 2011** | Spain | Single-center | Prospective | Full article | 153 | 47 | 53.6 | 75.8 | 20.9 | 3.3 |  | 6.33 | 100.0% | 1 | 153 | 0.10 |  |  |  |
| **El Braks, 2007** | France | Multi-center | Retrospective | Full article | 113 | 54.1 | 61.1 | 61.1 | 15.8 | 100.0 | 100.0 | 7.70 | 32.7% | 1 | 37 | 0.35 |  |  |  |
| **van der Meer, 2012** | The Netherlands | Multi-center | Prospective | Full article | 530 | 48 | 69.6 | 67.7 | 17.5 | 100.0 |  | 8.40 | 100.0% | 7 | 192 | 0.43 |  |  |  |
| **El-Serag, 2016** | USA | Multi-center | Retrospective | Full article | 10738 | 53.1 | 95.3 | 53.6 | 13.5 | 14.4 |  | 2.80 | 100.0% | 100 | 10817 | 0.33 |  |  |  |
| **Kobayashi, 2017** | Japan | Single-center | Retrospective | Full article | 528 | 54 | 58.0 | 100.0 |  | 9.7 |  | 7.30 |  | 12 | 528 | 0.31 |  |  |  |
| **Toyoda, 2016** | Japan | Single-center | Retrospective | Brief report | 578 |  | 58.4 |  |  | 61.0 |  | 1.00 |  | 2 | 578 | 0.35 |  |  |  |
| **Innes, 2017** | Scotland | Multi-center | Prospective | Full article | 585 | 48.1 | 77.1 |  | 64.3 | 40.4 | 91.0 | 3.50 | 100.0% | 34 | 585 | 1.66 |  |  |  |
| **Ioannou, 2017** | USA | Multi-center | retrospective | Full article | 35871 | 52.4 | 96.6 | 70.5 | 11.2 | 11.6 |  | 9.15 | 33.4% | 2651 | 35871 | 0.81 |  |  |  |
| **Nagaoki, 2017** | Japan | Single-center | retrospective | Full article | 244 | 59 | 52.0 | 100.0 |  | 28.3 |  | 8.00 | 100.0% | 13 | 244 | 0.67 |  |  |  |
| **Li, 2018** | USA | Multi-center | retrospective | Full article | 3534 | 54 | 95.9 | 28.5 | 9.3 | 13.1 |  | 7.41 | 66.6% | 196 | 3534 | 0.75 |  |  |  |
| **Hsu, 2013** | Taiwan | national | Retospective | Full article | 457 | 50 | 57.8 |  |  | 81.6 |  | 4.90 |  | 18 | 457 | 0.80 |  |  |  |

**Table S4. Names of IFN studies included and characteristics of patients in these studies.**

| **Mean** |
| --- |
| **Median** |

**Notes on individual studies**

In Hagihara, GT1 refers to only GT1b infected patients

Kanogawa includes 137 untreated patients (demographics for these patients included in overall analysis)

In Sanefuji, time to HCC is only for the SVR group (6/26 patients). Recurrence rate only reported for SVR group

In Yamashita, GT1 refers to only GT1b infected patients

In Mazzaferro, used recurrence rates for HCV/HBV core ab positive as well as ‘pure’ HCV-infected subjects

In Kudo, GT1 refers to only GT1b infected patients. Maintenance IFN was given for median of 4.7 years.

In Tanimoto, GT1 refers to only GT1b infected patients

Shiratori, GT1 refers to only GT1b infected patients

Urabe included IFN-based treatment regimens but some patients received IFN/RBV + telaprevir/simeprevir

In Hung, 116/132 patients completed IFN treatment

In Aleman, baseline characteristics also include 48 untreated patients

In Van der Meer, 47% were hep B core ab positive

El Serag is VA, also 3.5% were co-infected with HIV

In Kobayashi, GT1 refers to only GT1b infected patients

In Hsu, 6.1% with Hep B and 0.4% HIV co-infections

Denom = denominator

GT = genotype

HBV = hepatitis B

HCV = hepatitis C

IR = incidence rate

Num = numerator

VA = Veterans Affairs
